# Improvements in dizziness and imbalance results from using a multi disciplinary and multi sensory approach to Vestibular Physical Therapy – a case study

**DOI:** 10.3389/fnsys.2015.00106

**Published:** 2015-08-06

**Authors:** Kim R. Gottshall, Pinata H. Sessoms

**Affiliations:** ^1^Physical Therapy Department, Naval Medical Center San Diego, San DiegoCA, USA; ^2^Physiological and Cognitive Operational Research Environment Laboratory, Warfighter Performance Department, Naval Health Research Center, San DiegoCA, USA; ^3^Department of Exercise and Nutritional Sciences, San Diego State University, San DiegoCA, USA

**Keywords:** CAREN, vestibular physical therapy, virtual reality, treadmill, traumatic brain injury, TBI

## Abstract

This paper discusses a case study of a 41-year-old active duty male service member who sustained head trauma from a motorcycle accident and underwent multidisciplinary vestibular physical therapy rehabilitation. He was initially treated with traditional physical therapy applications of treadmill walking and standing balance with some symptom improvements, but was not able to maintain a running speed that would allow him to return to full active duty status. Further treatment utilizing a Computer Assisted Rehabilitation Environment was performed in order to increase level of difficulty and further enhance function. This treatment is able to elicit vestibular deficits seen in the community as it requires subjects to walk and balance while performing tasks within a virtual scenario incorporating platform motion, visual surround and flow, and cognitive processing. After 6 weeks of therapy, twice weekly, improvements in clinical vestibular measures were observed as well as walking speed and patient confidence. The patient was able to return to full duty after treatment. This case study provides supportive evidence that multidimensional tasking in a virtual environment provides a safe but demanding form of vestibular therapy for patients needing more challenging tasks than those provided with traditional therapy techniques. Those persons requiring higher levels of performance before returning to full duty (e.g., pilots, special operators, etc.) may find this type of therapy beneficial.

## Introduction

Traumatic brain injury (TBI) can be incurred from a multitude of incident types, including blasts, vehicle collisions, sports, and falls that induce a physiological disruption of brain function. It can be manifested in a variety of ways including a period of loss of consciousness, loss of memory, and/or an alteration in mental state at the time of the accident. The US military treatment facilities have invested many resources at over 18 clinical sites towards the treatment of persons with TBI, which has become one of the signature injuries of troops in recent conflicts. Over 250,000 service members have sustained a TBI over the last 14 years, with 83% classified as a mild TBI or mTBI (Defense and Veterans Brain Injury Center [DVBIC], 2015). Besides those sustained in combat, mTBI also occurs commonly from sports injuries, motor vehicle accidents, and training. Traumatic brain injury (TBI) can result in vestibular impairments such as headache, vertigo, dizziness, and imbalance (Paloski et al., 1993; Reschke et al., 1998; Koppelmans et al., 2013). Identification and treatment of these symptoms may require a team of clinicians in order to most effectively treat the patient and return him or her to full duty or reintegration into the community (Morris and Gottshall, 2014). This includes testing with otolaryngology and treatment with various therapists capable of addressing specific deficits of the patient. At the Naval Medical Center San Diego (NMCSD), patients can utilize state of the art therapy tools such as an anti-gravity treadmill, climbing wall, and balance testing systems to assess, measure, and train balance, posture, strengths, and mobility deficits. Virtual reality systems also help to assess, train, and motivate patients during therapy. Scenarios and settings can be customized to the patient in order to target specific areas needing treatment. The systems used at NMCSD range from small force plates that provide biofeedback on a computer screen, to the large scale immersive Computer Assisted Rehabilitation Environment (CAREN), located at the nearby Naval Health Research Center (NHRC), that challenges patients with treadmill walking, platform motion, and visual flow.

## Materials and Methods

### History

This case involves a 41-year-old Caucasian male on active duty in the U. S. Navy. The patient, a Navy Senior Chief Petty Officer Hospital Corpsman, was referred to the NMCSD Physical Therapy Clinic, with vestibular disorder due to a motorcycle accident. The injury occurred on April 30, 2014, when he was hit by a car at an intersection at approximately 35 mph. He had a Glasgow Coma Scale of 15 (mild TBI) on admission to the hospital. Loss of consciousness was for an unknown amount of time but first memory was 4 days after the accident. He sustained a splenic laceration, small intracranial hemorrhage, multiple facial fractures and abrasions. No operative lesions were evident. Splenectomy occurred and the patient was discharged 4 days later.

The patient complained of headaches (rating of 6 out of 10, with 0 representing no pain and 10 representing worst pain possible), insomnia, dizziness with fast movements, and mild cognitive issues (memory and concentration). Headaches were relieved daily with Tylenol. Evaluation by Otolaryngology showed no spontaneous nystagmus, positional nystagmus, or horizontal nystagmus. The sensation of dizziness fatigued with repetition. The patient presented to physical therapy for evaluation 22 days after injury. Initial clinical vestibular assessments were run to measure balance performance and activity. Measures included the Dizziness Handicap Inventory (DHI), a self-report questionnaire that quantifies the impact of dizziness on daily life and is a tool used to help clinicians understand what particular activities are problematic for the patient. The Activities-Specific Balance Confidence Scale (ABC) was also administered to subjectively measure confidence and steadiness while performing specific ambulation tasks (e.g., walk in a crowded mall, get in and out of a car; Powell and Myers, 1995). A computerized dynamic posturography (CDP) (Neurocom Inc, Clackamas, OR, USA) Sensory Organization Test (SOT) was also performed to determine detriments in maintaining postural stability in stance when other sensory cues (i.e., visual, proprioceptive, or vestibular) are unavailable (Nashner and Peters, 1990). A Functional Gait Assessment (FGA) was administered (Whitney et al., 2009) to assess ability to perform tasks while walking (e.g., walking with eyes closed, ambulating backward). Computer in-vision tunnel testing of perception time, target following (TF), dynamic visual acuity (DVA), and gaze stabilization (GS) while seated were also performed (Gottshall and Hoffer, 2010). These tests assess impairments in a patient’s ability to perceive objects accurately while actively moving the head. Lastly, Cervical Joint Position Error Test (Cervical JPET) was performed to assess the cervicocephalic proprioception and neck reposition sense (ability to relocate the head back to center after rotation in transverse and sagittal planes).

Results of these initial tests were as follows:

• Dizziness Handicap Inventory: 18 out of 100 (with 0 representing no perceived handicap due to dizziness)
• Activities-Specific Balance Confidence Scale: 78 out of 100 (with 100 representing complete balance confidence)
• Posturography CDP SOT Score: 60 out of 100 with a reduced vestibular profile (failing score for condition 5: eyes closed on a sway-referenced support)
• Functional Gait Assessment: 16 out of 30 (with 30 representing complete ability to perform tasks) – patient considered a fall risk
• Cervical JPET deviations:
  ◦ Right = 4.5^′′^ R, 3.0^′′^ R, 2^′′^R
  ◦ Left = off R, 6.0^′′^ R, off L
  ◦ Up = 4.5^′′^ up, 1.0^′′^ L, 2.0^′′^ up
  ◦ Down = 6.0^′′^ up, 3.0^′′^ up, 2.0^′′^ down
• Perception Time: 20 msec
• Target Acquisition:
  ◦ Left: 290 msec
  ◦ Right: 290 msec
  ◦ Up: 290 msec
  ◦ Down: 290 msec
• Target Following: 15° per second
• Dynamic visual acuity (LogMar) loss:
  ◦ Right: 0.10
  ◦ Left: 0.30
  ◦ Up: 0.10
  ◦ Down: 0.10
• Gaze Stabilization Test: unable to complete.

This patient wanted to remain on active duty and pass the semi-annual Navy Physical Readiness Test (PRT) comprised of sit-ups, push-ups, and a cardiovascular activity (run, swim, bike, or elliptical). In order to do this he needed to increase his running speed to meet the run time standard for 1.5 mile run. The patient scored poorly in walking with vertical head motion, walking with tandem steps, and walking with eyes closed during clinical testing. The short-term goal of walking 20 feet with horizontal head motion without veering side to side was set for 3 weeks into therapy. The long-term goal of being able to perform the DVAT with no more than 0.2 logmar DVA loss was set for 6 weeks into therapy.

After the intake assessments and interview, it was concluded that vestibular rehabilitation would target vestibular ocular reflex (VOR) abnormalities, vestibular spinal reflex (VSR) deficits, and dynamic balance dysfunction. The patient gave written informed consent in accordance with the Institutional Review Board at the NHRC prior to participating in the study.

### Treatment

The treatment plan commenced at the NMCSD vestibular physical therapy clinic with a focus on increasing head movement, balance, and optical stimuli. The basis for the treatment rationale was the standard, documented physical therapy protocol for such an injury and the symptoms presented. The goal was to improve upon balance and ambulation and decrease dizziness, headache, and pain level. Traditionally based vestibular physical therapy was conducted for approximately 2 months, two times per week. Therapy sessions lasted approximately 30–45 min. The retraining exercises help to improve the coordination of body movement and organization of sensory information such as that from vision, balance, and proprioception. Exercises included treadmill walking and standing balance with eyes closed. A home exercise program was also administered involving similar exercises. These involved eye and head movements (both separate, together, and alternating) with a moving printed card; somatosensory exercises with arms crossed, eyes closed and head and weight shifts; and gait in different directions. The patient progressed and was able to stand with eyes closed unassisted, walk outdoors, and perform small bouts of running at slow speeds. However, the patient continued to demonstrate difficulties when walking and turning his head in certain directions and could not run for long periods of time without dizziness.

Since the subject was highly motivated and goals included passing the PRT (requiring running a minimum of 4.9 miles/hr or 2.2 m/s), treatment utilizing treadmill walking and multitasking was implemented. The patient was introduced to therapy techniques utilizing the CAREN (extended, Motekforce Link, Amsterdam, The Netherlands) located at the NHRC in San Diego. The CAREN, an immersive environment with 6 degrees of freedom motion platform and built-in instrumented treadmill, is ideal for assessment and rehabilitation of gait and balance disorders. Patients can interact with the system due to integrated force plates measuring weight shifting and body motion with an embedded motion capture system. Detailed visual displays are projected onto a 180° screen and move in synchronization with platform and subject movements. Immersion is further enhanced with realistic sounds and scents. Numerous biomechanical measurements are recorded, unobtrusively, as patients engage in the CAREN applications. Subjects are secured in a full body safety harness attached to an overhead truss which allows them to move freely on the treadmill. Several applications have been used in conjunction with each other to create a therapy session incorporating physical and cognitive multitasking to simulate real world situations.

**FIGURE 1:**
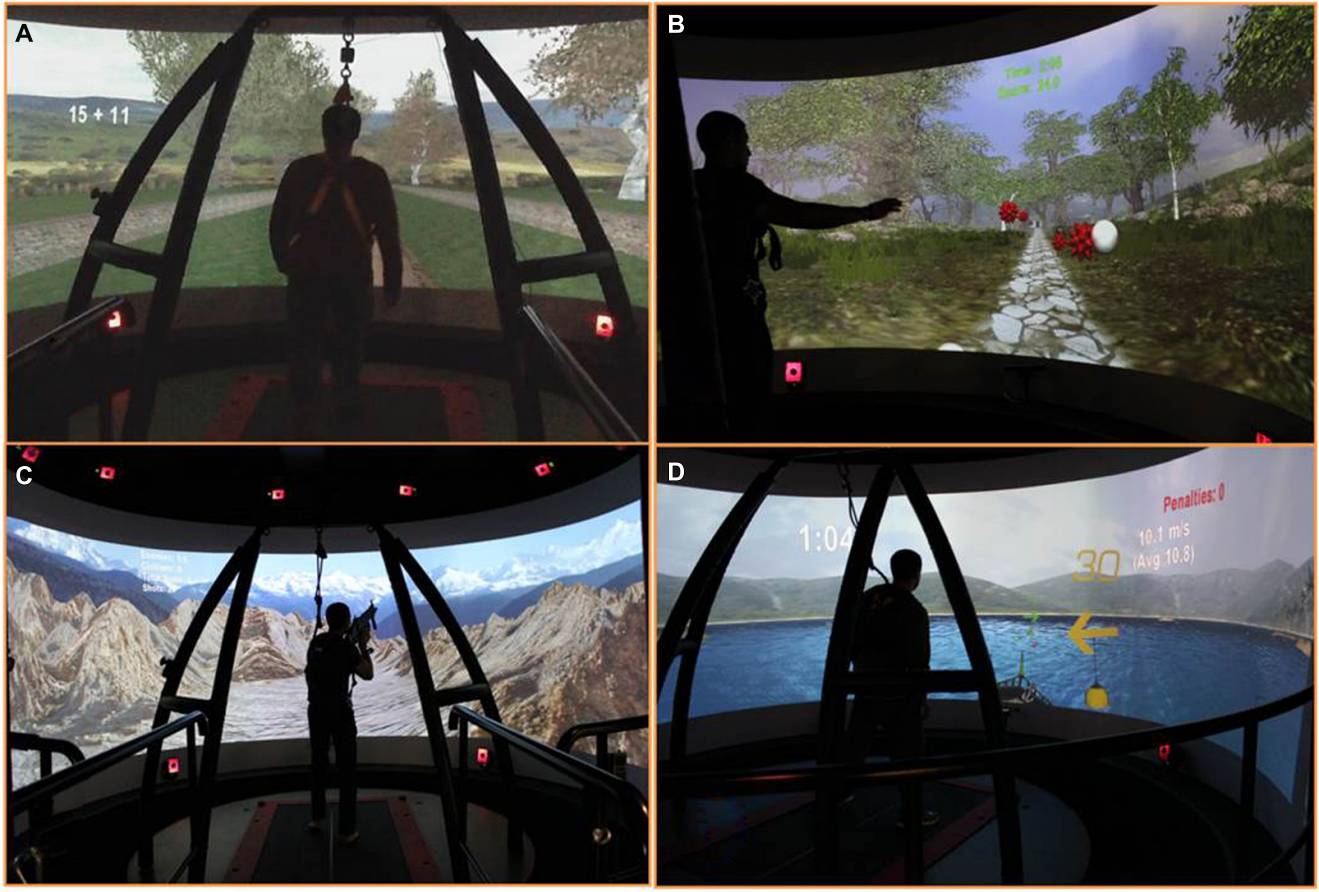
**Four different applications used for treatment of the patient on the Computer Assisted Rehabilitation Environment (CAREN). (A)** Endless Road, **(B)** Forest Road, **(C)** Mountain Patrol, and **(D)** Boat.

Treatment sessions on the CAREN lasted 45 min, once per week for a total of six weeks. The sessions included various immersive applications where the patient had to balance on the moving platform or walk while performing different physical or cognitive tasks (**Figure 1**). This included the “Endless Road” application where the patient walked on a straight path while performing a series of cognitive tasks, which were presented in order of: (1) direction of optotype letter “E”; (2) addition or subtraction of two numbers; and (3) stroop test (name of a color is displayed on the screen (e.g., “blue,” “green,” or “red”) with uncorrelated font color and the patient either says the word or color of the font). These tasks were given to provide a multitasking component to the activity as well as perform side-to-side head turning (scanning), as the images (numbers and words) were presented randomly on the left and right sides of the screen. The subject chose his own walking speed by pressing buttons to increase or decrease speed using a handheld game controller. After an initial “warm-up” round of the three cognitive tasks, the same tasks were performed again but with the addition of platform motion. This included a platform gyration during the optotype testing, left or right tilt when the math equations were presented, and a left or right shift when the stroop word was presented.

The next application, “Forest Road,” consisted of the subject walking on a path with variable terrain. This included walking up and down hills and left and right curves in the path. The platform moved accordingly with the path terrain (platform tilts up and down or platform shifts left and right). At the same time, the subject was given a task of hitting moving objects within the scenery by swatting at them as they passed. An optical motion capture system (Motion Analysis Corp, Santa Rosa, CA, USA) picked up movement of his hands, allowing for interaction with the virtual environment. This multi-tasking application challenged the patient by making him visually follow a moving target while walking over uneven terrain. The patient had to anticipate, plan, and vary his posture and gait in order to successfully hit targets. A scoring system based on walking speed, amount of platform motion, and number of targets hit, engaged the patient and measured his progress over each session.

A third application, “Boat,” challenged the patient’s balance and visual processing as the subject was tasked to drive a boat through a slalom course using side to side shifting to control the boat’s direction left and right and fore-aft movement to control speed (Sessoms et al., 2015). The platform tilted and moved up and down, simulating the boat movement as it went over waves in the water. Platform motion scaling was increased over the visits, as the subject became more comfortable with the scenario. A scoring system based on average speed, number of buoys successfully passed, number of objects hit (counted as a penalty), wave height, and platform scale, allowed the patient to challenge himself and measure his progress over each session.

The fourth application used in the therapy was a “Mountain Patrol” where the subject walked through a simulated mountainous path and patrolled for enemy targets. Motion capture markers placed on an airsoft weapon that the patient carried was used to target and shoot within the virtual scenario. As the patient walked on the treadmill (speed set by the therapist), platform motion mimicked the terrain of the path. The multisensory inputs of moving objects (e.g., Mine-Resistant Ambush Protected (MRAP) vehicle passing by), visual flow, surround sound, platform motion, and targeting while walking made this a challenging task. As this was a longer scenario, the objective was to increase the amount of time that the subject performed the task, up to 10 min in duration, increase walking speed, and vigilance.

The same clinical measurements obtained during the initial assessment (i.e., DHI, FGA, etc.) were also obtained at the 3rd and 6th week of treatment on the CAREN to measure improvements in vestibular function.

## Results

### Results during Therapy

The patient progressed in walking speed and amount of platform motion (**Table 1**) that could be tolerated without losing balance throughout the CAREN sessions. Some applications only showed slight differences between sessions, specifically in comparing outcomes between sessions 3, 4, and 5. In some cases, small decrements were observed for one application, but overall, session improvements were observed. Specific data were recorded for each application on the CAREN. During the Endless Road application, the chosen walking speed of 1.22 m/s at the patient’s first visit was faster for this patient than that observed for the initial gait speeds of 0.6 m/s for a similar mTBI population walking on the CAREN (Sessoms et al., 2015). At each subsequent visit, the subject was able to maintain or increase his walking speed and amount of platform motion with no difficulties. The slower speed at Visit 4 may have been due to exercising or report of headache before therapy.

**Table 1:** Computer Assisted Rehabilitation Environment Application Measurements.

Application	Measurement	Visit 1	Visit 2	Visit 3	Visit 4	Visit 5	Visit 6
Endless road	Walking Speed (m/s)	1.22	1.49	1.53	1.45	1.68	1.74
	Average Platform Motion Scale	0.2	0.6	0.75	0.75	0.95	1.18
Forest road	Score	55.2	100.4	106.7^∗^	102.8^∗^	104.5^∗^	126.8
Boat	Score	369	557^∗^	612^∗^	666^∗^	775	936
	Average Platform Motion Scale	0.03	0.025^∗^	0.03^∗^	0.03^∗^	0.1	0.2
	Average speed (m/s)	4.3	5.9^∗^	6.4^∗^	7.1^∗^	7.7	8.7
Mountain patrol	Time on application (min:sec)	0:00	3:21^∗^	7:40^∗^	8:15^∗^	10:10	8:42^†^
	Walking speed (m/s)	N/A	1.15^∗^	1.08^∗^	1.10^∗^	1.20	1.30

*Measurements of performance taken during CAREN therapy over the visits. ^∗^denote the application and visit where patient had balance issues or stumbled. ^†^denotes the shorter time compared to previous visit because the patient walked faster and reached the end of the scenario.*

In the Forest Road application, the patient’s score increased by over 200% between the first and last session. The patient experienced some difficulties in the third and fourth sessions, maintaining a steady gait when walking uphill with the larger platform motions. Treadmill speed (set by the therapist) was thus reduced when walking uphill for these sessions until the patient was able to maintain a consistent gait. By the last session, the patient was able to maintain his balance and consistent gait throughout the application and thus achieve a higher score by Visit 6.

**FIGURE 2:**
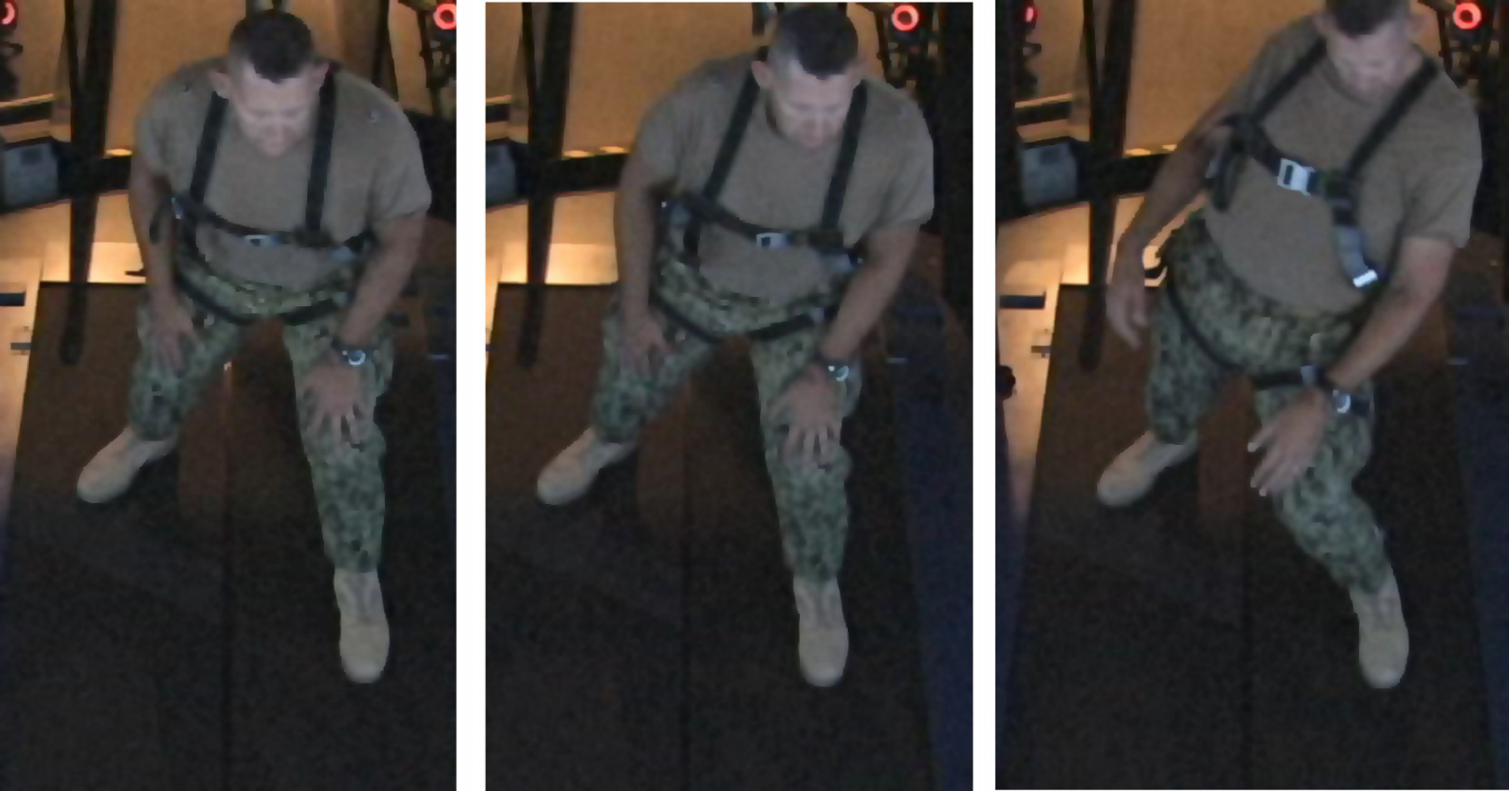
**Patient becoming unstable when difficulty (amount of platform motion) in the application increases.** By the end of 6 weeks of treatment, subject was able to perform all applications without instability.

During the Boat application, the patient continually improved his score from session to session, due most likely to the increased platform motion at each session and his ability to maintain a higher average speed each time. The patient initially displayed some balance difficulties (**Figure 2**) throughout the sessions as platform motion and his average speed increased, but did not show any problems with balance by Visit 5.

During the Mountain Patrol application, the patient stumbled during the first several visits as visual stimuli passed him on his left side or when he moved his head down to engage a close target located on the path in front of him. The total time on the application was thus kept relatively short during Visit 2 because of these symptoms. The patient, aware of these provoking factors, was able to double his time on the application by Visit 3 and by Visit 5, the subject was able to walk a full 10 min without any adverse symptoms. Walking speeds were similar between Visits 2–4, but was fast enough by Visit 5, that the subject was able to finish the scenario (i.e., walk to the end of the path). The subject was able to finish the scenario without difficulties in Visits 5 and 6.

### Clinical Results

**FIGURE 3:**
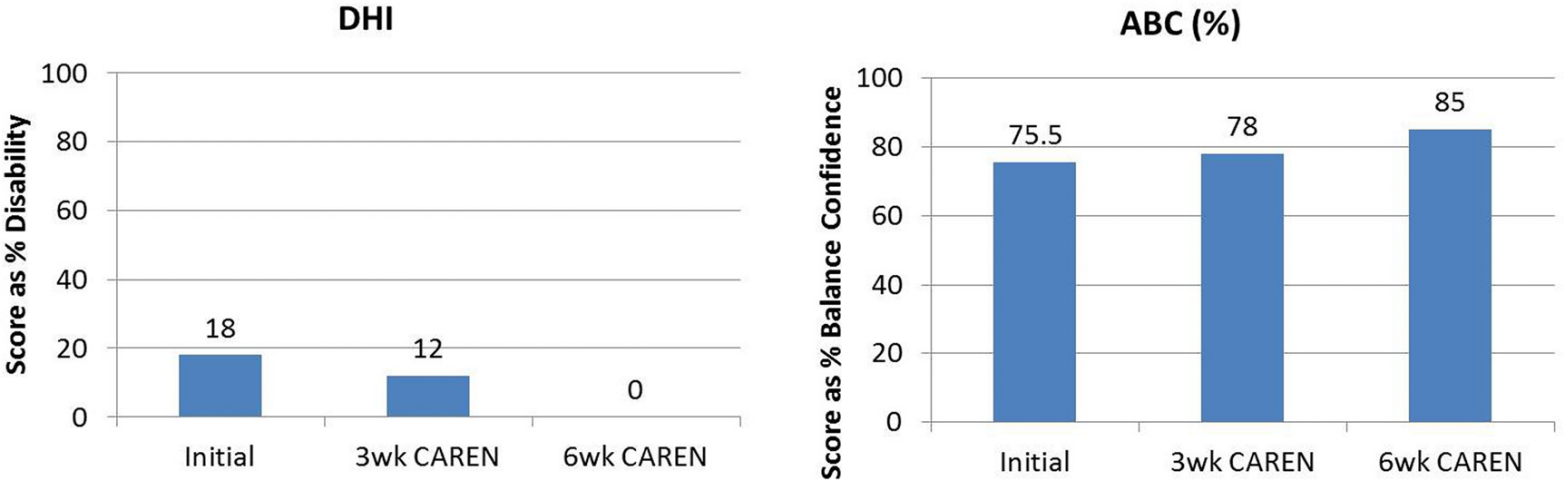
**Subjective questionnaire outcomes of Dizziness Handicap Inventory (DHI) and Activities-Specific Balance Confidence Scale (ABC) scores of the patient at the three measured time points: (1) initial intake, (2) after 3 weeks of CAREN therapy, and (3) after 6 weeks of CAREN therapy**.

**FIGURE 4:**
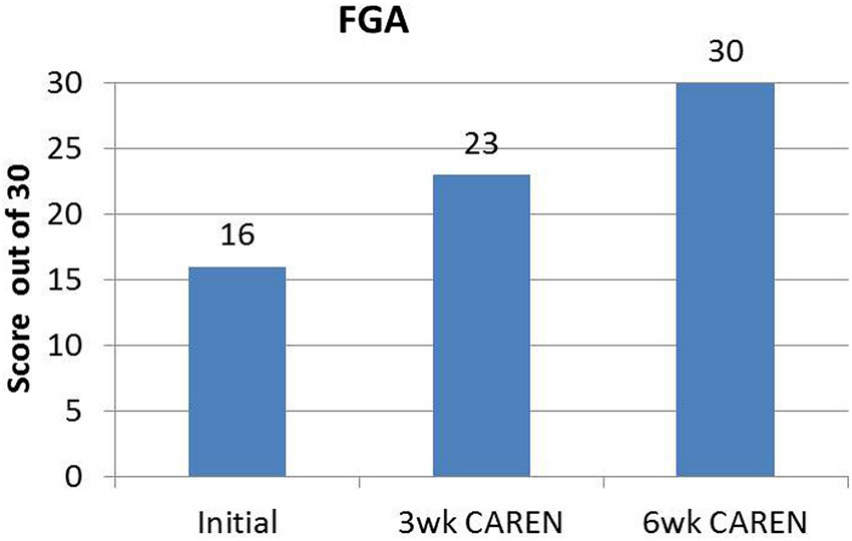
**Functional Gait Assessment (FGA) scores of the patient at the three measured time points.** By 6 weeks of CAREN therapy, the patient reached the maximum value of 30 for the test.

**FIGURE 5:**
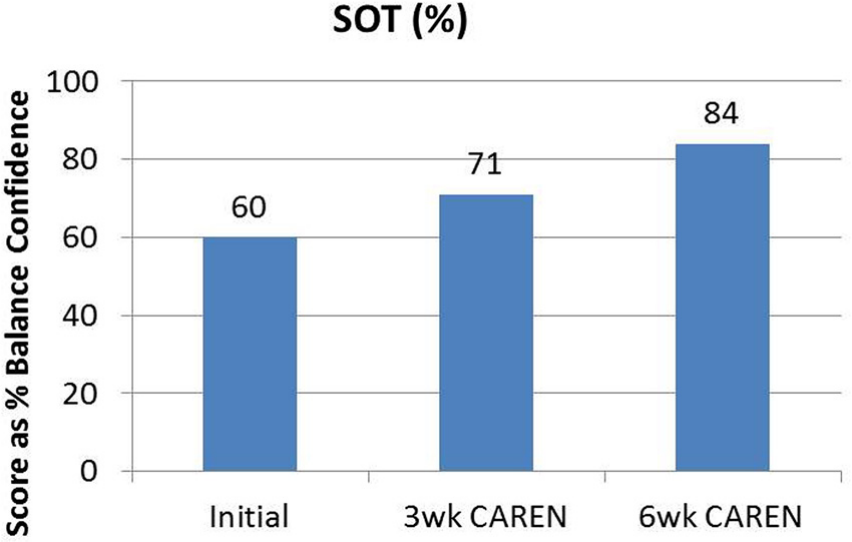
**Sensory Organization Test (SOT) scores of the patient at the three measured time points.** By 6 weeks of CAREN therapy, the patient fell within normal healthy limits.

**FIGURE 6:**
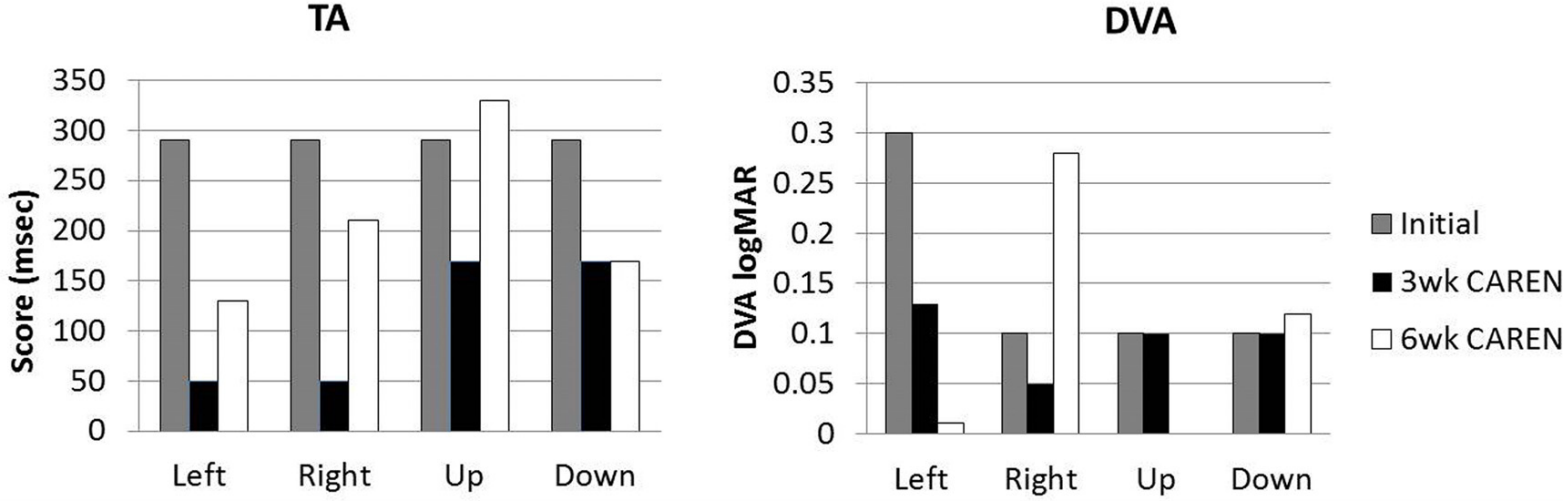
**Target Acquisition (TA) and Dynamic Visual Acuity (DVA) test scores at three measured time points**.

**FIGURE 7:**
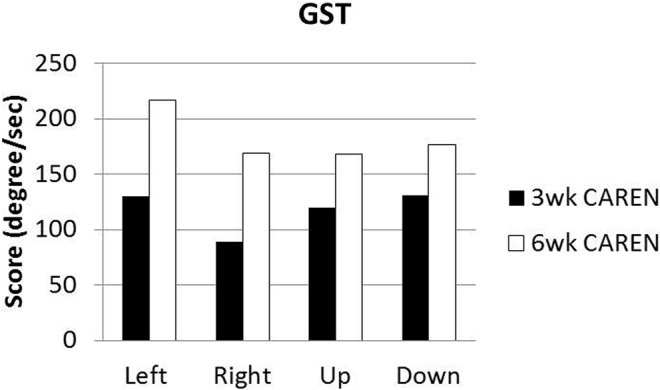
**Gaze Stabilization Test (GST) scores at 3 and 6 weeks time points.** Subject was unable to complete GST test at the initial assessment.

Re-assessments of all the initial measurements showed improvements in function as therapy sessions progressed. The DHI score decreased to 0 (no dizziness) by Week 6 of CAREN treatment (**Figure 3**) and ABC scores consistently increased over the 6 week period. The patient’s FGA score also increased over time, reaching the maximum of 30 points (**Figure 4**) by Week 6. Additionally, the High-level Mobility Assessment Test (HiMAT; Williams et al., 2005), which the subject was unable to perform initially, was performed at Week 6. This included high-level activities such as running, jumping, hopping, and stairs. A score of 53 out of 54 was obtained, where 54 was the best score possible. The SOT score came within normal limits by Week 6 (**Figure 5**). Overall, TA, DVA, and GST scores improved compared to initial assessments (**Figures 6** and **7**).

The patient had met short and long term goals set at the initial evaluation. Walking speeds during CAREN therapy increased from 1.2 to 1.7 m/s. Stamina improved, as length of walking time on the CAREN also increased at each session and ability to walk and balance with increasing motion platform improved. The patient reported being able to run faster and longer distances in the community and actions known to elicit dizziness were reduced after treatment. The subject was able to pass the Physical Readiness Test and remains on active duty status.

## Discussion

This patient required a complex, multidisciplinary approach to care in order to return to a high level of function after TBI. Spatial orientation requires the coordinated integration of the somatosensory, visual, and vestibular systems. Therapy utilizing an immersive multisensory virtual environment can help to challenge the patient in therapy and challenge the vestibular system in a similar way to that required in the community. The treatment on the CAREN allowed the patient to identify movements that elicited the symptoms that sometimes occurred in the community but could not normally be replicated in traditional physical therapy.

The challenges to dizziness and balance affecting this patient are similar to those experienced by astronauts readapting to the gravitational environment. When astronauts return from spaceflight, they must improve their ability to integrate their visual, vestibular, and proprioceptive senses to maintain balance and vestibular function. Therapy using a system similar to the CAREN may show benefits to astronauts who see decrements in performing functional tasks and ambulation. The ability to provide cognitive challenges while walking through a virtual environment allows them to better integrate all somatosensory senses within a safe environment and has the potential to speed up the process of improving spatial disorientation back to a level of high functional performance (Shumway-Cook and Woollacott, 2000; Pellecchia, 2005). Besides post flight rehabilitation, there may also be benefits for the use of a similar system for preflight training.

Management of the patient with vestibular dysfunction and treating symptoms that are sometimes hard to elicit, requires a team approach to get the most effective therapy possible. Clinicians (doctors, nurses, and therapists) must work together with case managers, counselors, and family to decide the best avenue of care that will engage the patient and challenge them to the best of their abilities. Multiple techniques may have to be used in tandem or in conjunction in order to provide the ideal therapy for treatment of patients with unresolved vestibular dysfunction.

## Conflict of Interest Statement

The authors declare that the research was conducted in the absence of any commercial or financial relationships that could be construed as a potential conflict of interest.
